# The Role of Depression on Treatment Adherence in Patients with Heart Failure–a Systematic Review of the Literature

**DOI:** 10.1007/s11886-022-01815-0

**Published:** 2022-11-03

**Authors:** Valentina Poletti, Francesco Pagnini, Paolo Banfi, Eleonora Volpato

**Affiliations:** 1 Department of Psychology, Università Cattolica del Sacro Cuore, Milan, Italy; 2 Department of Psychology, Harvard University, Cambridge, MA USA; 3 IRCCS Fondazione Don Gnocchi, Milan, Italy

**Keywords:** Heart failure, Depression, Medication adherence, Adherence to treatment, Systematic review

## Abstract

**Introduction:**

Although poor medication adherence is considered an impacting risk factor for worsening heart failure (HF) outcomes, adherence rates in HF patients continue to be considerably low. To improve this condition, several studies investigated the impact of many determinants on medication adherence; however, few authors explored the role of depression on it.

**Purpose of Review:**

The purpose of this systematic review was to explore the association between depressive symptoms and medication adherence in HF patients. In particular, the research question was is depression a barrier to medication adherence in HF patients?

**Methods:**

A systematic review of quantitative analysis studies was undertaken. Six electronic databases were searched between the end of October and March 2022. Thirty-one trials were included, all of them assessed depression, adherence to medication, and their possible relationship.

**Results:**

As was intended, findings showed that the impact of a mild to moderate level of depression was significant on adherence to treatment in HF patients. However, many other risk factors emerged, like family support and health practices (es. low sodium diet).

**Conclusion:**

The detection of depression in the setting of HF should be crucial to HF patients’ physical health and quality of life. Future research should take depression into account, exploring this area through self-report and qualitative interview as well.

**Supplementary Information:**

The online version contains supplementary material available at 10.1007/s11886-022-01815-0.

## Introduction

Heart failure (HF) consists of the inability of the heart to supply the required amount of blood and oxygen to meet the peripheral tissues’ demands [1]. HF has two main ways of manifesting itself: it can develop suddenly (acute form) or over time as the heart gets weaker (chronic form) [2].

Worldwide, 64.3 million people live with HF and the prevalence of known HF is estimated at 1 to 2% of the adult population [3]. About 5.7 million people in the USA have HF [4], while in Europe, 14 million people suffer from it, with an incidence of 3.6 million new cases annually [1]. Moreover, since its incidence is associated with age, the prevalence of HF is expected to rise because of the ageing of society [5], leading to talk about “an emerging epidemic” [6].

The first classification of HF was proposed in the 2001 American Heart Association American College of Cardiology guidelines [7], with the intent of emphasising both the evolution and progression of the disease by defining 4 stages: A, B (preclinical stages), C and D (clinical stages) [8]. Afterward, patients were used to be classified according to the severity of symptoms and their impact on physical activity through the New York Heart Association (NYHA) Functional Classification [9], which places patients in four distinct categories, from the first, characterised by no limitation of physical activity, to the fourth, the most disabling [10]. Nowadays, left ventricular ejection fraction (LVEF, a phenotypic marker indicative of underlying pathophysiological mechanisms and sensitivity to therapy, is considered a more reliable clinical parameter. Therefore, patients are most often categorised as having HF with reduced (HFrEF,LVEF < 40%, mid-range (HFmrEF; LVEF 40–49%, or preserved ejection fraction (HFpEF; LVEF ≥ 50% [11].

The main symptoms that characterise HF include dyspnoea, elevated jugular venous pressure, tachycardia, or peripheral oedema [1]. Heart failure can also damage the liver and kidneys. Other complications include pulmonary hypertension or other heart conditions, such as an irregular heartbeat, and cardiac arrest [2].

As a consequence of these symptoms, HF has an extremely negative impact on the quality of life of patients and this leads to the implementation of some harmful coping strategies, like social avoidance or reduction of physical activity [12].

To improve the symptomatic condition and, consequently, patients’ quality of life, HF guidelines recommend a high level of adherence to prescribed medications, which corresponds to the extent to which a patient’s medication behaviour coincides with the prescribed medication regimen [13]. Despite this, medication nonadherence–which can manifest itself as underdosing, overdosing, drug holidays, or even taking medication that is not prescribed [14] – continues to be a common problem in HF patients (rates range from 10 to 93%, with most investigators citing rates of 40 to 60%) [13].

Medication nonadherence in HF is significantly associated with adverse events and impaired prognosis [15–17], and due to its impact, several systematic reviews investigated the role of many determinants of adherence.

The most common risk factors that emerged are the level of perceived social support [18], some healthcare system-related factors (institutionalisation, outpatients visits…), and several treatment-related factors (like dosing or continuation of therapy) [19, 20].

Beyond that, even some psychosocial factors, like depression, which is one of the most prevalent psychological complications in HF patients [21••], influence the prognosis of HF [22•].

Mbakwem et al. [23] demonstrated that one in five people with HF has depressive symptoms, with 48% of these having major depression and a meta-analysis of 28 studies found that depressed people were 46% more likely to develop cardiovascular disease than healthy people, concluding that depression appears to increase the risk of developing HF and vice versa [17].

In this regard, DiMatteo and collaborators [24] explored the role of depression in many medical conditions and reported a significant relationship between depression and noncompliance in different chronic diseases, including HF, with an odds ratio of 3.03 (95% confidence interval, 1.96–4.89).

This thesis could explain the reason why the development of depression in association with HF increases the risk of morbidity and mortality [25].

The continuously growing evidence about the role of depression on medication adherence and its increasing rate indicates the need for an integrative systematic synthesis of current data.

Despite all the statistical evidence, in fact, in literature, there is no recent review that explores this possible relationship or specifically focuses on depression as a clinical determinant for a low level of adherence to medication in a group of HF patients.

## Objectives

This systematic review aims to explore the association between depression, defined according to the Diagnostic and Statistical Manual of Mental Disorder (DSM-5), and medication adherence in patients who suffer from heart failure.

In particular, the main research question is as follows: Is the presence of depressive symptoms in subjects suffering from heart failure connected to a reduction in medication adherence compared to patients who do not have any depressive symptoms?

## Methods

A protocol was registered with PROSPERO international prospective register of systematic reviews (ID: CRD42021293445) on the 26th of December 2021.

This systematic review was conducted and reported in line with the Preferred Reporting Items for Systematic Reviews and Meta-Analyses (PRISMA) [26], considering the following phases: formulation of the research question and hypothesis, identification and selection of the relevant studies, data charting, collating, summarising, and reporting results.

### Inclusion and Exclusion Criteria

Original peer-reviewed articles produced in English and French from 2006 to 2021 were reviewed. A 15-year range was chosen to reflect the marked increase in multimorbidity literature in recent years. Opinion pieces, conference presentations, books, letters, and editorials were not reviewed.

Inclusion/exclusion criteria are reported in Table 1.

**Table 1:**
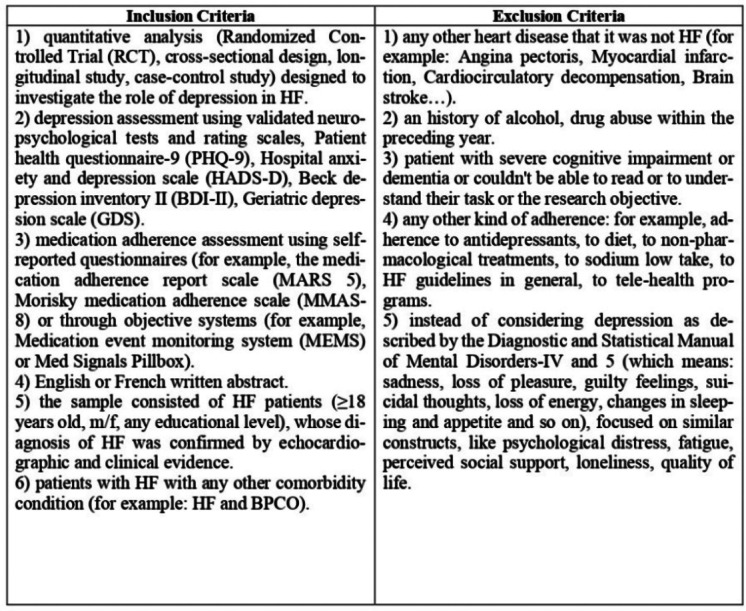
Inclusion and exclusion criteria

### Search Strategy and Study Selection

Six electronic databases were searched between the end of October and March 2022: PubMed, Scopus, Web of Science, PsycINFO, Cochrane Library, and Google Scholar. The following search terms were used with MESH terms and heading as relevant: depression, depressive symptoms, depressive disorder, major depressive disorder, drug adherence, medication adherence, medication non-adherence, medication nonadherence, adherence to treatment, compliance, non-compliance, patient compliance, heart failure.

Search strategy is reported in Table 2.

**Table 2:**
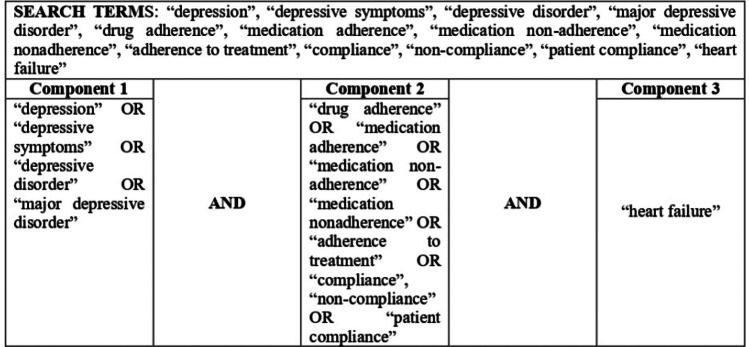
Search strategy

The references of the bibliographies of the included studies were considered to identify further studies. Review publications on associated topics were checked in order not to miss any important related articles.

All search results were pooled, and duplicates removed. Titles and abstracts were screened before analysing the full texts to decide their eligibility. The screening process was undertaken by two independent reviewers (VP and EV). Any disagreements were resolved and discussed with a third reviewer (FP), where needed. If more than one article was based on the same cohort, the report with the greatest number of participants was selected for data extraction to obtain prevalence estimates.

### Data Extraction and Management

A data extraction form was developed to extract relevant data from each included study and all extracted data were entered into RevMan 5.4.1. by one review author (VP) and checked for accuracy against the data extraction sheet by a second review author (EV) working independently. The two authors met to discuss data extraction and a third author (FP) was available to discuss any discrepancies.

Data were abstracted on general information: author, year of publication, title, journal, country, number of participants, study type, the language of publication; and patient information: diagnosis, age, gender, ethnicity, comorbidity.

Mean scores and standardised deviations of depression and medication adherence scores were extracted from each reviewed article. When available, we also reported the type of analysis used to compute the association or the relation between the two variables of interest (i.e. correlation, ANOVA, ANCOVA, regression, and multivariate regression). The statistical significance level was between *p* < 0.05 and *p* < 0.01.

Where studies reported medication non-adherence, this was converted to medication adherence by subtracting the number of non-adherent participants from the total sample. One reviewer (PV) extracted data from all included studies, and a second reviewer (VE) cross-checked 20%.

Information about reliability and validity of outcomes measure, ethical approval, and standardised protocol were annotated.

While extracting mean scores and DS, many missing data emerged from the articles (in particular, numerical scores of depression and medication adherence), so we decided to check the conclusions and look for any information about those variables.

Given the nature of the available data, a meta-analysis was not undertaken and instead a descriptive synthesis was used.

### Assessment of Risk of Bias

The quality of included studies was assessed using the version 2 of the Cochrane risk-of-bias (RoB 2) [27] for the randomised control trial; the Newcastle–Ottawa Scale (NOS) [28] for the case–control studies; finally, for the cross-sectional study, the NIH quality assessment tool for observational cohort and cross-sectional studies was adopted (Study Quality Assessment Tools, National Heart, Lung, and Blood Institute) [29].

These checklists included descriptive issues and internal and external validity. Assessment of quality was evaluated according to these published checklists by two independent authors (VP, EV), and doubts were clarified with the help of a third one (FP). No studies were excluded based on quality.

Outcomes are reported in the chart below (Results – “Quality Analysis” section).

## Results

### Overview of Studies

The literature search yielded 241 articles, after removing duplicates (81, 33.61%), 160 articles were reviewed (66.29%). Of these 160 articles, 129 were excluded (80.62%): the majority because they focused on a generic construct of “psychological distress”, holding “depression”, “fatigue”, and “stress” (*n* = 54; 41.86%), while, summarising many different articles, 37 (28.68%) of them were about “adherence” considering several aspects of that, for example adherence to physical exercise, salt diet, yoga, HF guidelines etc., but not specifically “medication adherence”. In the end, 31 articles are included.

**Fig. 1:**
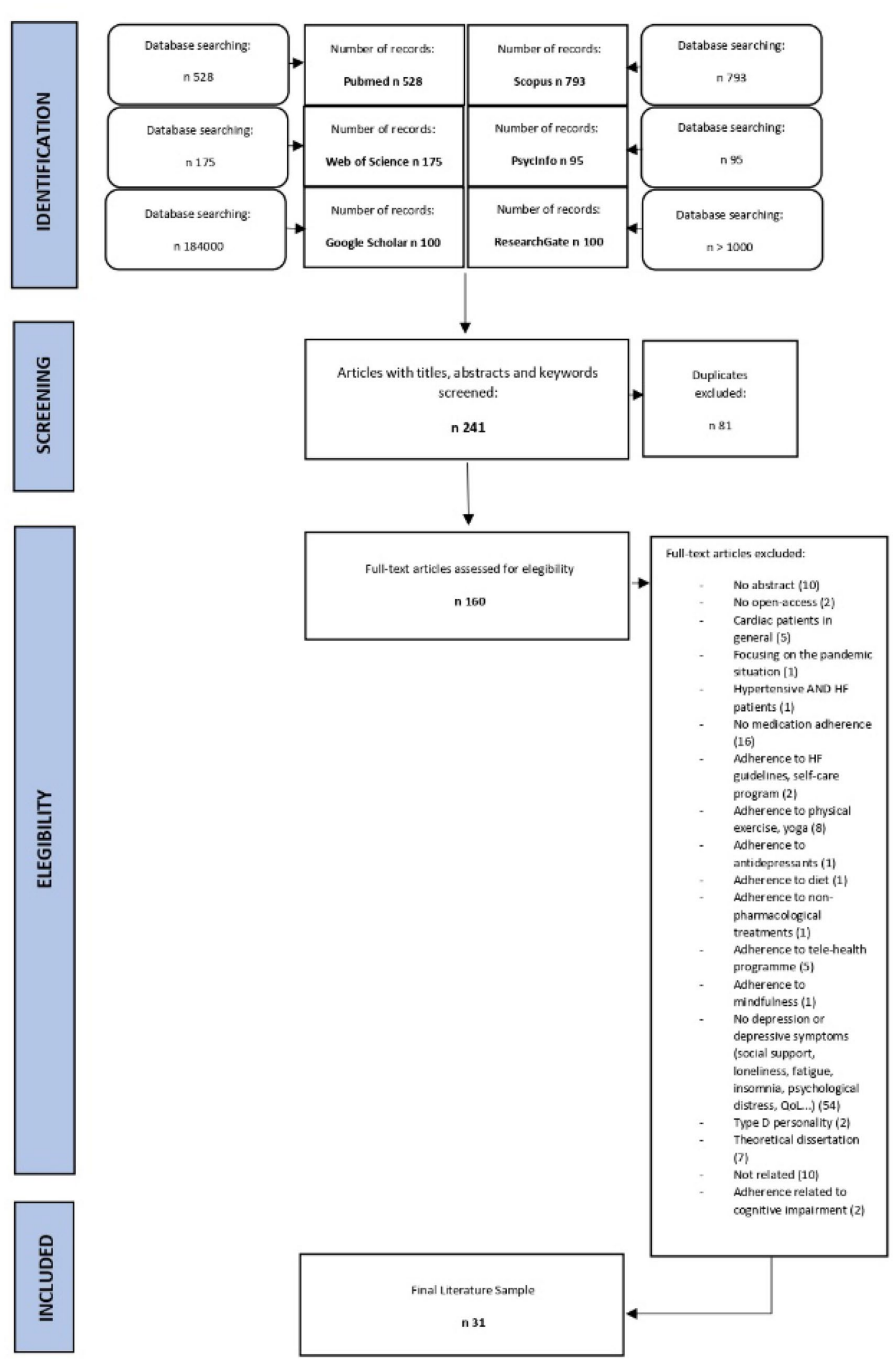
PRISMA flow diagram of the study selection process

The study selection process is reported in Fig. 1 (PRISMA flow diagram).

### Study Characteristics

Among the 29 studies, 16 were cross-sectional designs [30–45], followed by 7 longitudinal studies [35, 46–51], 2 case–control studies [52, 53], 1 prospective observational cohort design [54], 1 randomised control trial [55], 1 retrospective observational study [56••], and 1 quasi experimental study [57].

Patients were recruited from academic health care settings, heart failure clinics–cardiology departments, generic hospitals, and larger cohort studies.

Please refer to Supplementary Table 1 for the characteristics of the articles.

Regarding depression, we mainly selected those articles where the presence of depressive symptoms was assessed at the baseline. We chose to consider even those cases with major depressive disorder.

Talking about medication adherence, we considered both articles that used self-reports and objective tools, even if the literature asserts that self-report measures tend to overestimate medication adherence because of the social desirability bias [58], while electronic monitoring seems to be a more accurate tool to predict clinical outcomes [59].

Considering that only a few studies specifically focus on depression as a potential barrier to medication adherence, we also took account of those secondary variables which could be statistically significant (*p* < 0.05) or could play a role in the assessment of the two variables of our interest.

Please refer to Supplementary Table 1 for the secondary variables.

### Study Populations

A total of 516,244 HF patients were included in this systematic review. Patients were mainly from the USA (*n* = 16) [31–37, 44, 46, 47, 50, 51, 53–56••] followed by Iran (*n* = 3) [48, 57, 60], Brazil [30], Netherlands [38], Korea [42], Saudi Arabia [45], Ethiopia [43], Morocco [40], Denmark [49], and Australia [41]. Three articles did not specify their sample nationality [18, 39, 52].

The mean age of the sample was 63.99 ± 10.8 years old and about 50% were males. Following the eligibility criteria, people could show a comorbid condition: diabetes [30, 37, 38, 48, 56••], hypertension [38, 48, 56••], and COPD [30, 37, 38, 56••] were the most common.

Regarding the HF diagnosis, patients were mostly classified according to the NYHA Functional Classification (*n* = 21); classes II and III were more frequent than classes I and IV. Only three studies [37, 42, 52] classified their HF patients according to the LVEF parameters without considering the impact of symptoms on physical activity. In this case, many of the samples presented a reduced ejection fraction (HFrEF,LVEF < 40%). Finally, ten studies considered both the NYHA and the LVEF parameters [13, 30, 31, 36–38, 41, 48, 53, 60].

### Assessment of Depression

Among our sample, depressive symptoms are quite common (no difference between males and females). The prevalence of scores identified a mild to moderate level of depression: through the Patient Health Questionnaire-9 (PHQ-9) [61], the prevalence of scores were between 5 and 14 points [32, 50], and the same range was assessed with the Beck Depression Inventory (BDI-II) [31, 62] and the Hospital Anxiety and Depression Scale (HADS-D) [48, 63].

Moderate depressive symptomatology was also observed with the Center of Epidemiology Studies Depression Scale (CES-D) [64], with scores ranging from 17.60 (13.60) [36] to 19.83 (13.94) [33].

Finally, according to the Geriatric Depression Scale (GDS-30) [65] assessment system, the sample consisted of above 30% of depressed HF patients [35], but no numerical data are available.

### Assessment of Adherence to Medication

As previously explained, there are two main strategies to assess medication adherence: through objective systems or self-report scales [66]. We decided to consider both, but to keep results separate.

Starting from the objective systems, the authors used two principal tools:

- The Medication Event Monitoring System (MEMS) (*n* = 11)
- A med signal pillbox (*n* = 3)

The MEMS consists of an automatic compilation of times of medication intake, and it is considered a reliable way to predict drug concentration in plasma [67]. Through this tool, our sample was quite adherent to prescriptions (ranging from 66.8% [35] to 87% [38]).

On the other hand, a med signal pillbox is an electronic pillbox often used in chronic diseases, which allows monitoring of medication adherence continuously basis [53]. Dolansky et al. [54], Gathright et al. [53], and Goldstein et al. [34] all confirmed a good level of adherence to medication (about 73% of the sample).

Regarding the subjective assessment of medication adherence, a variety of different tools was taken in the exam.

According to the Morisky Medication Adherence Scale (MMAS-8) [68], the percentage of adherence was different: 78% for Hansen et al. [55], 30.66% for Shamsi et al. [60], and 51.8% for Eisele et al. [32].

A high level of adherence was also found by Farrell et al. [33], who used the Medication Adherence Scale (MAS) [69], by Tegegn et al. [43] through the (Revised) HF Compliance Questionnaire, a self-report tool that explores six health behaviours (appointment-keeping, medication adherence (93.2%), sodium restriction, fluid restriction, daily weighing, and exercise) [70], and by Ragbaoui et al. [40] through the CARDIA-questionnaire (83%). In particular, according to Ragbaoui et al.’s findings, 120 patients were taking their medication almost all the time (taking medication more than 90%), 3 patients most of the time (taking medication 75–90% of the time), and 24 patients less than half of the time [40].

On the contrary, Alvarez et al. [30] through the Repetitive Education and Monitoring for Adherence for Heart Failure (REMADE) [70] found a low score (16.2 ± 4.1).

Finally, Tang et al. [50], through the Basel Assessment of Adherence Scale (BAAS), Maeda et al. [36], through the MOS Specific Adherence survey [67], and Lin et al. [48], through the Medication Adherence Report Scale (MARS-5) [71], found a moderate level of medication adherence, in the latter case directly influenced by the eHealth literacy knowledge (*β* = 0.53; SE = 0.14; *p* < 0.001). According to a Likert scale questionnaire built ad hoc for this study [37], only 12.2% reported difficulty taking their medications.

### The Role of Depression on Adherence to Medication

#### Descriptive Analysis

In most of the studies that choose to conduct descriptive analysis, the level of depression was higher in the group of non-adherent people than in the group of adherent patients [37, 38, 45, 46, 54, 55]. According to Navidian et al. [57], adherence to medication is higher in non-depressed patients (49 ± 2.42) than in depressed patients (31 ± 2.76 *p* < 0.001) and the same conclusion was found by Wu et al. [44] and Tegegn et al. [43].

Different findings were discovered by Tang et al. [50],in fact, there was a significant difference between depressed and non-depressed participants in self-reported medication nonadherence (75% vs. 57% *p* = 0.008), but not in objectively measured medication nonadherence (28% vs. 33% *p* = 0.72). The depressed sample was 2.3 times more likely to self-report poor medication adherence than those who were nondepressed (*p* = 0.006).

#### Correlation

In most of the studies, the authors could observe that there was a statistically significant correlation between the two variables of our interest. In particular, they found a positive correlation between depression and non-adherence [33, 42, 56••] and a negative correlation between depression and adherence [31, 48].

Only Alvarez et al. [30] concluded that depression and adherence were not correlated (*r* = −0.12; *p* = 0.16).

According to Lindsay-Rahman et al. [18], only those participants who lived alone and with a high shined of depression showed a negative correlation between adherence and depression (*r* = −4.1855, *p* = 0.0021).

#### Regression

After adjusting for age, gender, ethnicity, marital status, education, comorbidity, and NYHA class, many different studies also found that there was a statistically significant relationship between depression and adherence. Specifically, several surveys confirmed that the level of depression can influence the level of medication adherence in a group of HF patients [32, 36, 38–40, 43, 47, 53, 54, 60], while, according to Nouamou et al. [39], also non-depressed HF patients did not respect the time taken medication, not only the depressed group.

In particular, considering specific treatments, Rasmussen et al. [49] observed that a high level of depression was a predictor of non-adherence to ACEI, ARB, and ARN (adjusted odds ratio 1.04, 95% CI: 1.01–1.07, β-blockers: adjusted OR 1.05, 95% CI: 1.02–1.09, MRAs: adjusted OR 1.06, 95% CI,1.01–1.11).

Moreover, talking about this relation, Chhabra et al. [72] discovered that depression modified the effect of medication adherence on hospitalisations (interaction term *p* < 0.001), too. Compared to high adherence, poor adherence was associated with a 24% increased hospitalisation rate among non-depressed Medicare beneficiaries with chronic HF and a 45% increased hospitalisation rate among depressed chronic HF. This relation between depression-adherence-hospitalisations was also confirmed by Johnson et al. [35].

On the other side, Goldstein et al. [34] noticed that this relation was mediated by the medication regimen complexity, in fact: in the group of patients with higher levels of depression, more regimen complexity was associated with lower adherence, while, for individuals with lower or average levels of depressive symptoms, regimen complexity was unrelated to medication adherence.

Schweitzer et al. [41] found that depression failed to predict adherence; however, we will not discuss this finding because there is no data available.

### Secondary Variables

Medication adherence is not the only advice the HF guidelines give [7].

Several authors [31, 32, 35, 40, 43, 47, 57] also assessed adherence to self-care practices, including questions that directly measure behaviours associated with fluid and weight management, low-sodium diet, and health behaviours (influenza vaccination, number of physicians contacts, physical activity).

Overall, participants showed low scores in all these areas; in particular, according to Tegegn et al. [43] only 28% had overall good adherence to self-care practices and most patients had a higher level of poor adherence to weight monitoring (87.6%), regular exercise (85.6%), and fluid restriction (70.5%).

According to the European heart failure self-care questionnaire and the self-care behaviour questionnaire (30), mean scores indicated a middle-low level of adherence to self-care practices [40].

Moreover, as Biddle et al. [47], Eisele et al. [32], Navidian et al. [57], and Tegegn et al. [43] discovered, depression influenced all the scores,in fact, non-depressed HF patients were 2.5 times more likely adherent to good health practices than depressed patients [43].

Depression and anxiety played a role in adherence to self-care guidelines and medication adherence, too. For example, according to Biddle et al. [47], there was a negative relationship between anxiety and adherence to self-care (*β* = −1.048; *p* = 0.014) and the same relationship was confirmed by Eisele et al. [32] (*β* = −0.117; *p* = 0.05) and by Lin et al. [48] (*β* = −0.27; *p* < 0.01).

Regarding medication adherence, Dolansky et al. [54] and Wu et al. [13] discovered that the level of anxiety of the adherent group was a little bit lower than in the non-adherent group (12.4 ± 5.1 vs. 13.6 ± 5.5,0.54 ± 0.57 vs. 0.80 ± 0.82). However, there was no association between anxiety and medication adherence [54]. The same thesis was confirmed by Rasmussen et al. [49], in fact, according to the HADS symptoms of anxiety did not show any association with nonadherence in any analyses.

Several authors also decided to focus on the level of perceived social support.

Some of them [36, 40, 54] noticed that the level of perceived social support was higher in the adherent to medication group than in the non-adherent group and the relationship between adherence and depressive symptoms could be mediated by living arrangement (*p* = 0.0324) [18]. On the other side, according to Farrell et al. [33], there was no correlation between adherence to medication and social support (*r* = −0.129; *p* > 0.05).

Regarding the level of social support, an important role was played by family: Chung et al. [52], for example, focused on patients’ marital status: people with a spouse were 3.1 times more likely to be adherent (took > 85% of medication doses as prescribed) to medication taking than patients without a spouse (95% CI = 1.06–9.0) and the strength of the relationship between marital status and adherence was equal to that between depression and adherence (odds ratio = 3.2,95% CI = 1.02–9.8). The same finding was observed by Chung et al. who concluded that having a spouse [52] was a predictor of a better level of adherence.

Other important predictors of a good level of treatment adherence were living in a rural place [51], spirituality [30], lower level of self-efficacy [32, 36], and patients’ personal beliefs [30, 46].

### Quality Analysis

According to the quality checklists, we decided to include all the 31 studies we selected. Nevertheless, we reported that some articles, for example Ragbaoui et al. [40], Chhabra et al. [72], Schweitzer et al. [41], So et al. [42], and Zeineddine et al. [45], did not define the criteria for inclusion clearly. Moreover, Zeineddine et al. [45] and So et al. [42] did not identify any confounding variable.

Regarding the quality analysis conducted with the Review Manager, we noticed that the biggest risks of bias were the performance and the detection bias, which means that personnel and participants were not blinded as well as the outcome assessors. Moreover, considering that only one study was an RCT study, we could also observe a high rate of risk in the selection bias.

Finally, we decided to report a level of high risk of bias in the “other bias” option: every single article we considered reported a list of the main limitations. Specifically, the most common limitation is the sample size, often small; the choice to use a self-report tool to assess the medication adherence (social desirability bias [66]) and the measurement time in the longitudinal studies (often short, like 21 days [54], 1 [46] or 3 months [47]).

**Fig. 2:**
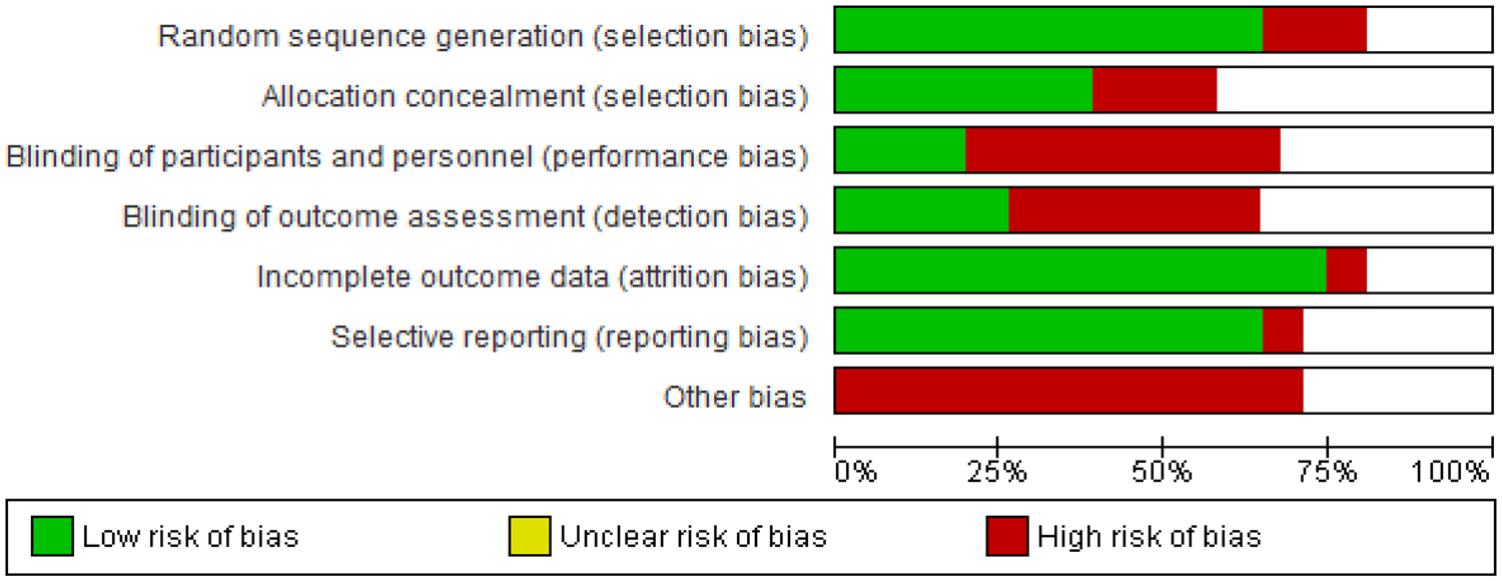
Risk of bias graph: review authors’ judgements about each risk of bias item presented as percentages across all included studies

The risk of bias graph is reported in Fig. 2.

**Fig. 3:**
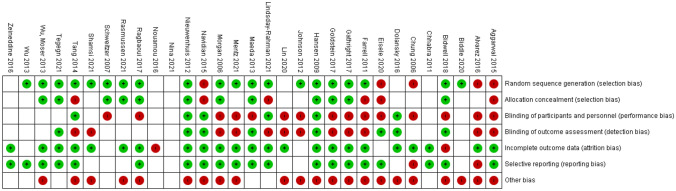
Risk of bias summary: review authors’ judgements about each risk of bias item for each included study

The risk of bias summary is reported in Fig. 3.

## Discussion

The need of investigating the role of depression in HF patients has grown from the exigence of a comprehensive assessment of well-being even in a clinical context, where patients’ psychological conditions seem to be less important than the physical ones.

As Geest et al. [73] explained, in fact, mental illness, like depression, can impact patients’ willingness to care and depressive symptoms, like fatigue or lack of motivation, are considered negative predictors of patients’ nonadherence to medication [24].

For these reasons, intending to take a first step toward overcoming a dualistic vision of the mind–body relationship, we synthesise the research assessing the effect of depression on patients’ adherence to their medical recommendations.

Every step was conducted, considering that medication adherence in HF is an essential part of a larger process, which consists of implementing several HF-related practices, such as taking a low sodium diet, keeping physically active, monitoring for symptoms of fluid retention by body weight measurement, and limiting excess fluid intake [74].

The first results that emerged from our sample were in agreement with the literature [21••]: many HF patients showed a mild to moderate level of depression and this comorbid condition confirmed the need to consider, among the various determinants of medication nonadherence, the role of patients’ mental health.

However, despite the prevalence of depressive symptoms, as reported by Mbakwem et al. [23], a severe classification of depression continues to be unusual, and this finding could be a consequence of the decision to avoid including patients with a diagnosis of major depressive disorder.

According to Hansen et al. [55], nonadherence is overestimated by people with depression, so, this removal could have altered the overall average of our sample.

Another issue that could alter the mean score of depression was the use of different tools to assess depression, so even the parameters to give an overall estimate were different from each other.

That systematic review discovered that the BDI-II and the HADS-D showed the best sensitivity and negative predictive values for detecting depression in cardiac patients, while, following this order, the BDI-II, the HADS-D, the CES-D, and the GDS-15 best-captured depression changes after cardiac rehabilitation delivery [75].

Despite these specifications, as the literature suggested [73], the presence of depressive symptoms [43, 47, 57] hurts self-care behaviour, awareness, and attitude and the same conclusions were found for anxiety [32, 48].

According to Celano et al. [76], there is also evidence that anxiety is associated with unhealthy behaviours and poorer adherence to physical activity or smoking cessation. Otherwise, according to Rassmussen et al. [49], some symptoms of anxiety, like a growing sense of alarm and danger, can lead patients to focus more on adherence to healthcare recommendations, including medication.

Focusing on medication adherence, our findings only partially agreed with the literature [13].

For example, those authors who used the MEMS system found that 66.8% [35] and 87% [38] of patients were adherents to medications, even if, in literature, rates range from 10 to 93%, with most investigators citing rates of 40 to 60% [13]. Scores were similar [34, 53, 54], with the med signal pillbox. According to Alvarez et al. [30] with the REMADHE system, the medication adherence score was low.

These findings suggested the need for a general clarification: again, the authors used several types of tools to analyse adherence and each tool focused on different aspects as well.

For example, the MEMS is a method of estimating when and how much a drug is administered [77], while the REMADHE tool is a self-report questionnaire composed of ten questions involving the use of medications and the respect of medical appointments from patients’ point of view [30].

However, discrepancy was noticeable even using the same tool, in fact, the percentage of adherence were different from each other even using MMA-S 8: 78% for Hansen et al. [55], 30.66% for Shamsi et al. [60], and 51.8% for Eisele et al. [32].

Moreover, we should also consider that medication adherence was assessed both with objective and subjective system. As literature reported [66], we also found many discrepant outcomes assessing the same sample [50, 55, 60].

Tang et al. [50] noticed that there was a significant difference between depressed and non-depressed participants assessed with self-report questionnaire (*p* = 0.008), but not in objectively measured medication nonadherence (*p* = 0.72).

In particular, the depressed sample was 2.3 times more likely to self-report poor medication adherence than those who were nondepressed (*p* = 0.006) and these results confirm that depression can play a potent role on patients’ perceptions of their behaviour.

This discrepancy between these two main systems may be explained by the fact that both target essential components of medication adherence such as dosing, timing, frequency, and forgetting; however, the self-reported adherence also captures the individual’s perceived ability to perform self-care, whereas the objective measure records the behavioural construct only [50].

On the other side, differences with the same tool can be caused by differences in the sample, for example we discovered that the perceived social support [18, 40, 52] is a predictor of medication adherence, and, often, patients who are not married are even less adherent to their therapy.

As Maeda et al. [36] explained, HF patients which perceive a high level of social support tend to perceive a larger diversity of external sources which can give them verbal encouragement, informational influence, and opportunities to observe and improve their disease management behaviour, increasing their levels of self-efficacy.

### Final Results and Findings

In conclusion, trying to answer our research question (Is the presence of depressive symptoms in subjects suffering from heart failure connected to a reduction in medication adherence compared to patients who do not have any depressive symptoms?), we can partially agree on the starting hypothesis.

Generally, HF patients who suffered from depression or showed depressive symptoms would be nonadherent to their prescribed medication treatment or, at least, less adherent than the non-depressed group.

In particular, depression was higher in the group of non-adherent people than in the group of adherent patients and vice versa [37, 38, 45–47, 54, 55, 57].

For example, Dolansky et al. [54] could observe that the depression score in non-adherent patients was 5.3 ± 5.2, while in the adherent group was 3.8 ± 4.5 (*p* < 0.05).

On the other side, Hansen et al. [55] found that, in the usual care group, the mean adjusted self-reported adherence was 75% for depressed participants and 81% for nondepressed participants (*p* = 0.04).

Moreover, according to our findings, depression and non-adherence to medication were positively correlated [32, 33, 36, 42, 43, 52, 56••], as well as depression and medication adherence were negatively correlated [18, 31, 48].

So, as the level of depression increased, the patient’s non-adherence also increased and vice versa.

The same correlation was found by Chhabra et al. [78], who also discovered that adherence has an impact on hospitalisation rates, too: compared to high adherence, poor adherence was associated with a 24% (95% CI 1.20–1.28) increased hospitalisation rate among non-depressed and a 45% increased hospitalisation rate among depressed.

Lastly, some authors [35, 40, 54, 60] identified a statistically significant relationship between depression and adherence, where depression was a predictor of adherence behaviours in HF patients.

To explain this connection, Rassmussen et al. [49] concluded that symptoms of depression might be characterised by impaired motivation and loss of initiative which potentially led to a negative impact on medication adherence and the same justification was given by Shamsi et al. [60] who discussed the role of motivation in treatment and its worsening in the depressed group.

According to Gathright and collaborators [53], changes in medication adherence and depression over time may contribute to changes in mortality risk, but there is no significant relationship between the two variables. According to Goldstein, this relationship may be mediated by the complexity of the therapeutic regimen [34].

However, not all the studies we considered confirmed our hypothesis: according to Alvarez et al. [30], for example, depression was not correlated to adherence, while Schweitzer et al. concluded that depression was not a predictor of adherence to medication [41].

### Strengths and Limitations

Even if there is a wide literature describing depression in HF, papers are often published in different speciality medical journals (for example cardiology, psychiatry, or nursing); therefore, sample sizes are always modest, and as consequence, results do not achieve good external validity. As consequence, a strength of our study is the possibility to make some inferences about depression and medication adherence, starting from a big sample of HF patients. Another important strength of the review is the confirmation of a statistical relation between depression and medication adherence, even if the sample was very heterogeneous.

On the other hand, this systematic review has some limitations.

The first one is the extreme heterogeneity of the sample patients suffer from different classes of HF and not all the studies used the same way to classify it. Moreover, comorbid conditions are different, and this could lead to an external validity problem.

Another important limitation concerns the tools that were used to assess variables of our interest. As explained before, even if most of the studies used validated tests, few studies used the same tools, so it is difficult to compare all the results; furthermore, not all the studies decided to report all the means and the standard deviations, and this is the reason why we could not try to make a meta-analysis.

Finally, another important limitation focuses on the construct of medication adherence itself, which had been assessed in two main different ways, the self-report and the objective system, that, even in the same study, showed discordant outcomes. This confirms the hypothesis that some biases must be taken into account.

### Clinical Implications: Recommendations for Evaluation and Therapy

In HF patients, a diagnosis of depression or the manifestation of depressive symptoms is highly prevalent, and this comorbid condition has been linked to poor functional outcomes. Our research confirms that HF patients with a greater propensity to have depressive symptoms are less adherent to medication and non-medication treatment, and this leads to a worsening of HF symptoms and, therefore, of patients’ quality of life.

The identification of the relation between depression and adherence to treatment favours the recognition of the need to introduce integrated therapeutic approaches, more focused on patient characteristics and less on disease in general.

For this reason, as Rasmussen et al. [49] discussed, person-centred care using patient-reported outcomes (PROMs) may carry a potential for identifying patients at increased risk of future medication non-adherence.

Moreover, clinicians who treat heart disease should be more vigilant in the detection of depression in the setting of HF, because patients’ mental health is crucial to their physical health, and it may contribute to decreasing HF mortality rates.

Finally, these findings suggest that HF patients with depression may need an additional treatment before implementing self-management interventions, as the increased obligation of self-care may be too difficult for the depressed patient to manage [47, 57], considering the lack of motivation which impacts their quality of life.

An interesting suggestion could be introducing a screening questionnaire for depression during a regular cardiorespiratory monitoring, in order to implement a psychological support and a practical guide to those patients whose depression rate is over the clinical cut-off.

### Research Implications

Given the results that emerged, it would be interesting to investigate the dynamics between depression, as described by DSM 5, and adherence to treatment, through RCT studies, taking into account secondary variables, such as anxiety and perceived social support, which, in turn, have statistically significant interactions with treatment adherence. Moreover, to avoid any bias associated with self-report tools, it could be useful to use only objective systems, such as the MEMS, to verify the level of medication adherence and select only one type of depression tool to make easier comparisons between different findings.

## Conclusions

Depression and non-adherence represent potentially modifiable risk factors for poor medical outcomes. Future research is needed to understand how an integrated intervention can improve both outcomes.

## Supplementary Information

Below is the link to the electronic supplementary material.

Supplementary file1 (DOCX 46 KB)

## Abbreviations

BAAS: Basel Assessment of Adherence Scale
BDI-II: Beck Depression Inventory II
CES-D: Center for Epidemiologic Studies Depression
DSM 5: Diagnostic and Statistical Manual of Mental Disorders 5
GDS: Geriatric Depression Scale
HADS: Hospital Anxiety and Depression Scale
HF: Heart failure
HFrEF: Heart failure with reduced ejection fraction
LVEF: Left ventricular ejection fraction
MARS-5: Medication Adherence Report Scale
MEMS: Medication Event Monitoring System
MMAS-8: Morisky Medication Adherence Scale
NOS: Newcastle-Ottawa Scale
NYHA: New York Heart Association
PHQ-9: Patient Health Questionnaire
PRISMA: Preferred Reporting Items for Systematic Reviews and Meta-Analyses
PROMs: Patient-reported outcomes
QoL: Quality of life
REMADHE: Repetitive Education and Monitoring for Adherence for Heart Failure
