# Supplementary material for: The Role of Depression on Treatment Adherence in Patients with Heart Failure–a Systematic Review of the Literature
Source: Curr Cardiol Rep. 2022 Nov 3;24(12):1995–2008. doi: 10.1007/s11886-022-01815-0 (PMC9747824; doi:10.1007/s11886-022-01815-0)
Supplement: Supplementary file 1 — Supplementary file1 (DOCX 46 KB) [file 11886_2022_1815_MOESM1_ESM.docx]

| **ID** | **Journal** | **N (age)** | **Nation** | **Objective** | **Inclusion/exclusion criteria** | **Main Outcome(s)** | **Adherence to medication** | **Depression** | **Conclusion** | **Limitation** |
| --- | --- | --- | --- | --- | --- | --- | --- | --- | --- | --- |
|  | Journal of nursing education and practice | 20 | USA | To collect pilot data to inform the design of educational interventions  targeted to heart failure patients and their  caregivers to improve medication adherence | HF diagnosis, age >18 y.o.  Participants  were excluded if they were unable to  complete the interview and/or surveys,  or if they did not speak English or Spanish. | Factors associated with non-adherence: forgetfulness (50%),  having other medications to take (20%), being symptom-free (20%).  Dyads showed interest in mobile applications and text messagingbut not for medication-dispensing technologies. | Morisky Medication Scale | BDI-II | Enthusiasm from patients and  caregivers in new technologies  to aid in adherence was tempered by potential burden, and should be  considered when designing interventions to promote adherence. | Small sample size; patients were limited to NYHA  class II-III;  Medication adherence self-report. |
| Alvarez et al. 2016 | Arquivos brasileiros de cardiologia | 130 | Brazil | To assess whether spirituality may influence  adherence to management of  HF patients | HF diagnosis, age >18 y.o.  Exclusion criteria were inability to understand the study protocol  and to answer the questions without assistance due to cognitive impairment or auditory deficit. | Neither depression nor religiosity (assessed separately) was correlated  to adherence.But, the combination of spirituality, religiosity and personal beliefs was an  independent predictor of adherence when adjusted for demographics,  clinical characteristics and psychosocial instruments. | REMADHE | PHQ-9 | Spirituality, religiosity and personal beliefs were the only variables consistently associated  with compliance to medication in a cohort of outpatients  with heart failure. Our data suggest that adequately addressing these  aspects on patient’s care may lead to an improvement in  adherence patterns in the complex heart failure management. | Cross-sectional design; in Brazil spirituality and religion are very notoriously  important values |
| Biddle et al. 2020 | The Journal of Rural Health | 349 | Kentucky, Nevada, California | To identify predictors of adherence to HF self-care recommendations | HF diagnosis, age >18 y.o.  Patients were excluded if they were enrolled in an HF disease management program,  had a terminal illness, or had a neurological condition with impaired cognition. | Of the covariates tested in the regression model, being a male  (p=.009), having less anxiety (p=.018), not being depressed  (p=.017), and having higher perceived control (p=.003)  were predictors of improved self-care score. | European Heart Failure Self-Care Scale questionnaire | PHQ-9 | Data suggest interventions designed to promote adherence behaviors  should include an assessment of gender, anxiety, depression,  and perceived control for optimal outcomes. | Rural population sample, which may  limit the generalizability of the findings; the measurement time points were relatively short-term; |
| Bidwell et al. 2018 | Heart & Lung | 228 | Southeastern United States | 1) configurations  of shared HF knowledge in patient-caregiver dyads;  2) quantify the relationship  between configurations and patient self-care adherence  to dietary sodium and HF medications. | HF diagnosis, age >18 y.o.  Patients were  excluded if they had HF secondary to an untreated condition (e.g.  hyperthyroidism, untreated tachyarrhythmias), a recent myocardial  infarction (past 6 months), angina, imminent cardiothoracic surgery,  cognitive impairment or major psychiatric condition (as  documented in the medical record and by report), or uncorrected  vision or hearing impairment that would preclude participation | Two dyadic knowledge configurations: “Knowledgeable  Together” and “Knowledge Gap”. Dyads were more likely to be in the  “Knowledgeable Together” group if they were white and more highly educated, if the patient  had a higher ejection fraction, fewer depressive symptoms, and better autonomy support, and if  the caregiver had better quality of life. In unadjusted comparisons, patients in the “Knowledge  Gap” group were less likely to adhere to HF medication and diet. In adjusted models,  significance was retained for dietary sodium only. | MEMS | BDI-II | Dyads with higher shared HF knowledge are likely more successful with select self-care adherence behaviors. | Small sample  size |
| Chhabra et al. 2011 | Value in Health | 151,924 |  | 1) evaluate impact of comorbid depression on HF medication use and  adherence among Medicare HF users;  2) examine relationships between HF medication adherence and outcomes;  3) determine depression impact | HF diagnosis, age >18 y.o. | A higher proportion of depressed patients was hospitalized (76% vs. 63%,p<0.001)  and re-hospitalized (40.5% vs. 38.6%,p<0.001) compared to non-depressed.  In multivariable models, depression modified the effect of adherence on hospitalizations  (p<0.001). |  |  | Poor EBM adherence was associated with increased hospitalization in both  depressed and non-depressed groups, with a greater effect among depressed CHF_MED_BEN. |  |
| Chung et al. |  | 92 |  | To examine the impact  of patients’ marital status on adherence to medication. | HF diagnosis, age >18 y.o. | MEMS: patients took 87% of prescribed doses; only 67% of  medication doses were taken on time. No difference in age, education,  number of prescribed medication, or depression between patients with and without a spouse. Compared to patients without a spouse,  patients with a spouse took more prescribed doses (p<.05). In a test of a multiple logistic regression model consisting of marital status,  depression, and NYHA functional class, patients with a spouse were 3.1 times  more likely to be adherent  to medication taking than patients without a spouse.  The strength of this relationship was equal to that between depression and  adherence (odds ratio = 3.2; 95% CI = 1.02 − 9.8). | MEMS | BDI-II | Married patients had substantially better adherence to medication than patients without a spouse regardless of depression level and NYHA class. | More subjects than expected had atrial  fibrillation or paced cardiac rhythm;  Small sample size; sample lacks racial diversity. |
| Dolansky et al. 2016 | Circulation: Heart Failure | 309 | USA | to examine the impact of  cognitive function on medication adherence among  community-dwelling patients with HF | HF diagnosis, age >18 y.o. | In unadjusted analyses, lower scores on all 3 cognitive domains (executive functions, memory, attention)  predicted poorer medication adherence (p=0.001). After adjusting for demographic,  clinical, and psychosocial variables, memory continued to predict medication adherence, executive function and attention were no longer a predictor. | MedSignals Pillbox | PHQ-9 | Future studies should examine the link from cognitive impairment and medication nonadherence to clinical outcomes (eg, hospitalization and mortality). | 21 days was relatively short  and may have resulted in the high levels of adherence  observed because of measurement reactivity; Not  asking patients to keep a diary detailing reasons for missing certain doses given potential measurement reactivity of  the diary; |
| Eisele et al. 2020 | Family practice | 3099 | USA | to investigate the association between psychological  distress and adherence to medication and lifestyle recommendations | HF diagnosis, age >18 y.o.  Patients who have died since the last consultation, suffered from dementia  or were not regular patients of the surgery were excluded. | Psychological distress was significantly associated with poorer medication adherence.  Male sex, younger age,  lower self-efficacy and less familiar relation with the general practitioner were  common factors associated with both lower medication and lifestyle adherence. | MMAS-8 | PHQ-9 | Promising factors for increasing medication adherence (reduction of psychological distress) and lifestyle adherence  which were found in this cross-sectional study, must be further investigated in longitudinal studies. | not checking for cognitive impairment;  no validated instrument to measure lifestyle  adherence was available in German |
| Farrell et al. 2011 | Journal of clinical psychology in medical settings | 105 | Southeastern United States | To determine whether depression, hostility, and personality based Millon Behavioral Medicine Diagnostic Medication Abuse scale were associated with medication adherence  beyond contributions of demographic, medical, and psychosocial variables | HF diagnosis, age >18 y.o.  Patients were excluded if they had current drug or alcohol abuse,  current diagnosis of HIV or other immune disorder, current  treatment for cancer, or any psychiatric or cognitive disorder that would impede ability to complete questionnaires | In hierarchical regression, greater Medication  Abuse scale scores were associated with poorer adherence  above and beyond both depression (p=.037) and hostility (p=.014).  The Medication Abuse scale also completely mediated  the relationship between adherence and depression. | MAS | CES-D | These findings suggest that personality measures such as the MBMD and hostility scales might be utilized in future studies investigating predictors of adherence and also used clinically to predict medication adherence among HF patients. | Use of a self-report  adherence measure as the single measure of adherence  rather than or in addition to objective adherence measures, |
| Gathright et al. 2017 | Health Psychology | 308 | USA | to determine the relation between medication non-adherence, medication regimen and depression | HF diagnosis, age >18 y.o.  Individuals were  ineligible if they had cardiac surgery within 3 months prior to enrollment, had history of  neurological disorder or injury,  moderate or severe head injury, past or current significant psychiatric disorders,  renal  failure requiring dialysis, untreated sleep apnea, current substance abuse or within the past 5  years, or were currently using a home telehealth HF monitoring program | Depression was associated with  increased all-cause mortality risk, (HR: 1.87; 95% CI: 1.04 – 3.37).  Depression was not related to cardiovascular mortality, potentially  due to a low number of cardiac-related deaths. When medication  non-adherence was added to the model, non-adherence (HR: 1.01; 95% CI: 1.004 – 1.02),  but not depression, predicted all-cause mortality risk. | MED signal pillbox | PHQ-9 | Depressive symptoms confer increased all-cause mortality risk in heart failure, and medication non-adherence contributes to this relationship. Depression and non-adherence  represent potentially modifiable risk factors for poor prognosis. Future research is needed  to understand whether interventions that concomitantly target these factors can improve outcomes. | The mortality rate in the current study was  lower than expected; |
| Goldstein et al. 2017 | Journal of behavioral medicine | 299 | Ohio | To assess depressive symptoms as a moderator of the relationship  between medication regimen complexity and  medication adherence | HF diagnosis, age >18 y.o.  Exclusion criteria included class IV HF, a history of  neurological disorder that produces cognitive impairment, history of significant psychological problems | The interaction of medication regimen complexity and depressive  symptoms predicted medication adherence, p<.05. For individuals with  higher levels of depressive symptoms,  more regimen complexity was associated with lower adherence. For individuals with low or average levels of depressive symptoms,  regimen complexity was unrelated to medication adherence. | MED signal Pillbox | PHQ-9 | Care management strategies, including pillboxes and caregiver involvement, may be valuable in HF patients with depression. | The pillbox selected for this  study could only measure four medications; |
| Hansen et al. 2009 | Journal of the American Pharmacists Association | 314 | Indiana and North Carolina | To assess the influence of depression on adherence with  HF medications and to determine whether the effect of a pharmacy-based  intervention to improve HF medication  adherence is modified by depression | HF diagnosis, age >18 y.o. | At baseline, 37% of participants were depressed.  In the usual care group, mean adjusted self-reported adherence was  75% for depressed participants and 81% for nondepressed participants  (p=0.04); mean adjusted adherence measured electronically was 71%  for depressed participants and 69% for nondepressed participants (p=0.65).  Intervention effectiveness did not differ for depressed compared with nondepressed participants. | MEMS-Morisky Compliance Assessment Scale | GDS-15 | The effectiveness of a pharmacy-based intervention to improve adherence does not appear to  be influenced by depression. However, the method used to measure adherence influences the interpretation of the relationship between depression and heart failure medication adherence. | Small sample size; measurement of adherence and depression: Hawthorne effect  also might exist (i.e., a change in behavior as a result of being monitored);  The timing of each measurement: the electronic adherence  reflected average adherence during the 12-month study period, while self-reported adherence reflected the average  reported adherence at months 0, 6, and 12. |
| Johnson et al. 2012 | Journal of Cardiac Failure | 784 | USA |  | HF diagnosis, age >18 y.o.  Patients were excluded if they were classified as NYHA functional class I, or were unlikely to undergo or benefit from the behavioral treatment  (ie, presence of cognitive dysfunction or psychologic comorbidity); | Depression was a strong predictor (p=.006)  after adjusting for physician adherence to evidence-based medication use,  patient adherence to HF drug therapy, patient adherence to salt restriction,  illness severity, HF severity and socioeconomic factors. | MEMS | GDS-30 | Depression is a strong psychosocial predictor of repeated hospitalizations for HF. This finding suggests that clinicians should screen for depression early in the course of HF management. | Self-report data |
| Lin et al. 2020 | European Journal of Cardiovascular Nursing | 238 | Iran | To use a longitudinal design to examine the temporal associations between eHealth literacy,  insomnia, psychological distress, medication adherence and cardiac events among older HF patients | HF diagnosis, age >18 y.o.  Patients were excluded if they had intellectual disability or  cognitive impairment (as assessed using the mini-mental status exam; MMSE <20),  severe psychiatric problems, severe renal failure, untreated anaemia,  an unstable cardiac condition within the past 3 months | eHealth literacy had direct and indirect effects  on medication adherence and quality  of life. Moreover, eHealth literacy had protecting effects on cardiac events, through the mediators  of insomnia, psychological distress and medication adherence. | MARS-5 | HADS-D | As eHealth literacy was a protector for patients with heart failure, healthcare providers may plan effective  programmes to improve eHealth literacy for the population. Additional benefits of improving eHealth literacy in HF  may be decreased insomnia and psychological distress, improved quality of life, as well as decreased cardiovascular events. | Not heterogenous sample |
| Lindsay-Rahman et al. 2021 | European Journal of Cardiovascular Nursing | 208 |  | To determine whether perceived  social support and living arrangement moderated the association  between depressive symptoms and medication adherence | HF diagnosis, age >18 y.o. | Three-way interaction (depressive symptoms*living arrangement*PSS)  was significant (p=0.0324). The effect of depressive symptoms on medication  adherence was only significant for two groups: the living alone group  with high PSS (p=0.0021), and the living with a non-spousal  family group with low PSS (p=0.0349). For these groups,  their depressive symptoms were inversely associated with medication adherence. | MEMS | PHQ-9 | These results suggest that living arrangement and perceived social support are factors to be considered in medication adherence when planning care for patients with depressive symptoms. |  |
| Maeda et al. 2013 | International journal of behavioral medicine | 252 | USA | To examine whether self-efficacy mediates the contributions  of social support and depression to treatment adherence | HF diagnosis, age >18 y.o.  Patients were excluded if they had severe  cognitive impairments, neurological disorders,  or psychiatric disorders that prevented them from understanding  instructions or if they were receiving treatment for other  life-threatening medical comorbidities  that confounded assessment of their health status | Self-efficacy mediated the associations of social support  and depression with treatment adherence after adjusting  for demographic  and medical  covariates. | The MOS Specific Adherence survey | CES-D | Self-efficacy explains the influence of social support and depression on treatment adherence and may be a key target for interventions to improve disease management and self-care behaviors in HF patients. | Cross-sectional design |
| Mentz et al. 2021 | Journal of cardiac failure | 353,642 | USA | To evaluate treatment patterns in patients with HFrEF, both with and without WHFEs | HF diagnosis, age >18 y.o.  Patients with heart transplants, left ventricular assistdevices,  adult congenital heart disease, or amyloid-osis  during the baseline period were excluded. | Black race, asthma, chronic kidney disease, and depression  were associated with nonadherence to medication. | MEMS |  | This study demonstrated underuse of GDMT for patients with HF with or without WHFEs. Although there was a treatment escalation within 3 months following WHFE, it was not sustained thereafter. | Not heterogenous sample |
| Morgan et al. 2006 | Journal of cardiac failure | 522 | USA | To evaluate the association between difficulty taking  medications, depressive symptoms, and health status | HF diagnosis, age >18 y.o. | Patients with difficulty taking medications (n = 64; 12.2%) had worse health status (p=.008) and more depressive symptoms (43.8% vs 27.1%; p=.006). | Patient-reported difficulty taking  medications was quantified using a  5-level Likert scale question. | MOS-D | Among HF patients, difficulty taking medications is associated with worse health status. This association appears to be explained,  in part, by coexistent depression. | Cross-sectional design |
| Navidian et al. 2015 | PloS one | 70 | Iran | To evaluate the effect of self-care education  on awareness, attitude, and adherence to self-care behaviors | HF diagnosis, age >18 y.o.  Patients with difficulty in hearing and speaking,  having less than one week to discharge program, physical disability,  dialysis, and previous participation in rehabilitation programs were excluded. | After educational sessions, the statistical analysis showed  significant differences in the mean scores of awareness,  attitude, and adherence to self-care behaviors between the tdepressed and non-depressed (p<0.0001). | Self-Care Behaviors Questionnaire | BDI-II | Self-care behavior education had lower effects on the depressed HF patients. Therefore, before providing education for these patients, it is necessary to consider their psychological problems. | Lack of control group without any educational intervention, low sample size,  history of hospitalization, impact of previous self-care trainings, and short-term education  during hospitalization |
| Nieuwenhuis et al. 2012 | Netherlands Heart Journal | 37 | Netherlands | To describe differences in self-reported  and objectively measured medication adherence by the  MEMS based on an evidence-based cutpoint in a HF population  and to assess differences between adherent and non-adherent patients | HF diagnosis, age >18 y.o.  Exclusion criteria: discharge to a nursing home, withdrawal from COACH,  presence of end-stage HF or another terminal illness | All patients reported they ‘always’ took their  medication as prescribed.  However, when measured by the MEMS, only 76 % of  all patients were adherent. Non-adherent patients more  often had a complex medication regimen (p<.01),  more often depressive symptoms (p=.04)  and a shorter history of HF (p=.04), compared with adherent patients. | MEMS, Revised HF Compliance Questionnaire | CES-D | Medication adherence measured by the MEMS was remarkably lower than self-reported adherence. Given the evidence of its importance, further efforts are needed to improve adherence to the pharmacological regimen in HF patients. | Small sample size |
| So et al. 2021 | Korean Journal of Adult Nursing | 107 | Korea | To determine the levels of medication  adherence in patients with HF and explore the factors related to it. | HF diagnosis, age >18 y.o. | Medication adherence positively  correlated with depression (r=.34, p=.001) and barriers  (r=.48, p=.001) but negatively correlated with attitude  (r=-.39, p=.001) |  |  | To improve medication adherence, a nursing strategy to reduce long-term adverse effects and barriers accompanied by continuous monitoring is required. |  |
| Nouamou et al. 2016 | Archives of Cardiovascular Diseases Supplements | 147 |  | To examine relationships between depression measures  in explaining medication adherence in elderly HF patients | HF diagnosis, age >18 y.o. | 11% of depressed patients and 7,3% of non depressed  patients don’t respect the time taken medication. The relationship  between depression and non adherence persisted after adjustment  for potential confounding variables, including age,  education and social support (CI=95%, p=0.008). | MMAS-8 | PHQ-9 | Our findings should direct attention toward depression as independent predictor of medication adherence in elderly HF patients. |  |
| Ragbaoui et al. 2017 | The Pan African Medical Journal | 147 | Morocco | To evaluate the adherence to medical treatment and to explore the factors that contribute  to the adherence to drug treatment in Moroccan patients with chronic heart  failure using the multidimensional adherence model of the WHO | HF diagnosis, age >18 y.o.  Exclusion criteria:  severe cognitive impairment; lack of patient’s consent to participate | The predictive factors  that significantly influence medication adherence were:  Depression (p=0.034), level of social support (p=0.03)  and medication taken by the patient himself (p=0.0001). | CARDIAquestionnaire (self-report) | PHQ-9 | The different strategies that act on the predictive factors could improve medication adherence. | Small sample size |
| Rassmussen et al. 2021 | European Heart Journal-Cardiovascular Pharmacotherapy | 1464 | Denmark | To determine whether health-related  quality of life, anxiety, and depression  were associated with long-term medication adherence. | HF diagnosis, age >18 y.o.  Patients were excluded if: were not able to understand written or oral Danish, had no civil registration  number or unable to participate due to severe illness such as unconscious or terminal illness | In adjusted regression analyses, lower health-related quality of  life (EQ-5D and HeartQoL) and symptoms of depression  (HADS-D) at discharge were associated with non-adherence. | Patients reported outcomes (obj) | HADS-D | Lower health-related quality of life and symptoms of depression were associated with nonadherence across HF medications at one- and three years  of follow-up. Person-centred care using PROMs may carry a potential for identifying  patients at increased risk of future medication non-adherence. |  |
| Schweitzer et al. 2007 | Journal of Cardiovascular Nursing | 115 | Australia | To test the hypothesis that depression, anxiety,  and self-efficacy are independent predictors of such adherence | HF diagnosis, age >18 y.o. | Depression  failed to predict adherence. Self-efficacy strongly predicted adherence behavior | Heart Failure Compliance Questionnaire (HFCQ) | BDI-II | Findings will assist cardiac nurses to prepare strategies to optimize adherence and quality of life while minimizing public health costs. | Self-report data |
| Shamsi et al. 2020 | Psychiatria | 150 | Iran | To determine the relationship between  medication adherence and affective temperaments in HF patients | HF diagnosis, age >18 y.o. | There was a significant relationship  between medication adherence and depressive (p=0.049), cyclothymic  (p=0.01), and irritable (p=0.01) affective temperaments. Only BDI-II score  (p=0.018) was identified as predictors of drug adherence | Morisky Medication Adherence Scale (MMAS-8) | BDI-II | Despite the statistically significant relationship between depressive, cyclothymic, and irritable temperaments and adherence, affective temperaments cannot be considered as a predictor of adherence in HF patients |  |
| Tang et al. 2014 | Clinical nursing research | 280 | Northeastern United States | To explore the association  between depression and medication adherence in HF patients. | HF diagnosis, age >18 y.o. Exclusionary criteria were terminal illness, history of recent alcohol or drug abuse, severe cognitive impairment  severe depression | There was a significant difference between depressed and nondepressed participants  in self-reported medication nonadherence (p=.008), but not in objectively measured  medication nonadherence (p = .72). The depressed sample was 2.3 times more likely  to self-report poor medication adherence than those who were nondepressed (p=.006). | BAAS - MEMS | PHQ-9 | These study findings highlight the importance of comprehensive assessment of a well-being even in a clinical context when the psychological condition is not the predominant focus. | Exclusion of severely depressed  patients |
| Tegegn et al. 2021 | Patient preference and adherence | 396 | Ethiopia | To assess adherence to self-care practices and associated factors among HF patients | HF diagnosis, age >18 y.o.  Patients who had followed less than three months and  who were too ill to respond to the questions were excluded. | Comorbidity (AOR: 1.62; 95% CI: 1.07–2.624), level of knowledge  (AOR: 3.58; 95% CI: 2.23–5.79) and depression (AOR: 2.45; 95% CI: 1.048–5.726)  were significantly associated with adherence to self-care practice. | Revised Heart Failure Compliance Scale | PHQ-9 | Comorbidity, inadequate knowledge, and depression were predictors of self-care practice. As a result, nursing intervention programs regarding knowledge  on HF are recommended for enhancing self-care practices. |  |
| Wu et al. 2013 | Journal of cardiac failure | 216 | Aardex-USA, Union City, California | [To explore the combined influence of medication  adherence and depressive symptoms for prediction  of cardiac event–free survival in HF patients](https://www.sciencedirect.com/topics/medicine-and-dentistry/event-free-survival) | HF diagnosis, age >18 y.o.  Patients were excluded if they had a  myocardial infarction or stroke within 3 months, obvious cognitive  impairment, were discharged to a skilled nursing facility, or were  diagnosed with a coexisting terminal illness. | The risk of experiencing a cardiac event for  patients with medication nonadherence and depressive symptoms was 5 times  higher than those who were medication adherent without depressive symptoms. | MEMS | PHQ-9 | Medication nonadherence and depressive symptoms had a negative synergistic effect on cardiac event–free survival in patients with HF. | Small sample size; HF severity assessed with the use of NYHA functional class instead of a more objective marker |
| Wu et al. 2010 | Heart and Lung: Journal of Acute and Critical Care | 136 | USA | To compare event-free survival between HF patients living in urban and rural areas. | HF diagnosis, age >18 y.o.  Patients were excluded if they had a serious  terminal illness or obvious cognitive impairment. | Rurality (p=.04) predicted event-free survival after controlling  for age, marital status, NYHA class, medications, medication adherence,  depressive symptoms, and social support. | MEMS | PHQ-9 | Rural patients were less likely than their urban counterparts to experience an event. Further research is needed to identify protective factors that may be unique to rural settings. | Not  heterogeneous sample |
| Zeineddine et al. 2016 | Journal of the Saudi Heart Association | 50 | Saudi Arabia | To determine the association between depression and  the medication adherence in HFpatients | HF diagnosis, age >18 y.o. | Depression has a major impact on the  medication adherence in HF patients (p < 0.001). | Morisky Medication Adherence Scale (MMAS-8) | PHQ-9 |  |  |

**Table 3** – Characteristics of included studies
